# Mucinous adenocarcinoma of the prostate with extremely low prostate-specific antigen levels, recurrent disease: a case report

**DOI:** 10.3389/fonc.2025.1645306

**Published:** 2025-08-14

**Authors:** Xu Yu, Jie Yuan, Zhiming Wang, Niannian Wang, Xiangfei He, Jie Liu

**Affiliations:** ^1^ Linyi People’s Hospital, Shandong Second Medical University, Weifang, Shandong, China; ^2^ Linyi People’s Hospital, Linyi, Shandong, China

**Keywords:** prostate cancer, mucinous adenocarcinoma, case report, pathology, therapy

## Abstract

Mucinous adenocarcinoma (MC) is a very rare type of prostate cancer, accounting for less than 1% of prostate adenocarcinomas, and its etiology is still unclear. Its clinical symptoms are similar to those of prostate hyperplasia and follicular carcinoma of the prostate, which are often misdiagnosed, and a definite diagnosis depends on pathological diagnosis, and the secretion of a large amount of mucus is its pathological characteristic. Surgery and endocrine therapy are the main treatment modalities, followed by radiation therapy and comprehensive therapy. Since this disease is rarely reported, in order to improve the understanding and diagnosis of clinically rare MC, we report a case of primary recurrent mucinous adenocarcinoma of the prostate and analyze its clinical features, pathological diagnosis, treatment and prognosis in the light of the literature.

## Introduction

Mucinous adenocarcinoma of the prostate, also known as glial adenocarcinoma, is a rare subtype of prostate cancer (1–6). Boyd described the first case of mucinous adenocarcinoma of the prostate in 1882 (7). In 1979, Elbadawi described and defined mucinous adenocarcinoma of the prostate. In common prostate adenocarcinomas, it is not difficult to see a small amount of mucinous material, but MC, which accounts for about 0.4% of prostate cancers (8), actively produces abundant mucinous material in which the tumor cells are dispersed in a lake of mucus. Due to the low incidence of prostate mucinous adenocarcinoma, this paper retrospectively analyzes the clinical data of one patient with prostate mucinous adenocarcinoma admitted from December 2019 to March 2025, from initial diagnosis to recurrence to follow-up, and explores the diagnostic and therapeutic features of the disease in combination with the relevant literature review, which is reported as follows.

**Figure 1:**
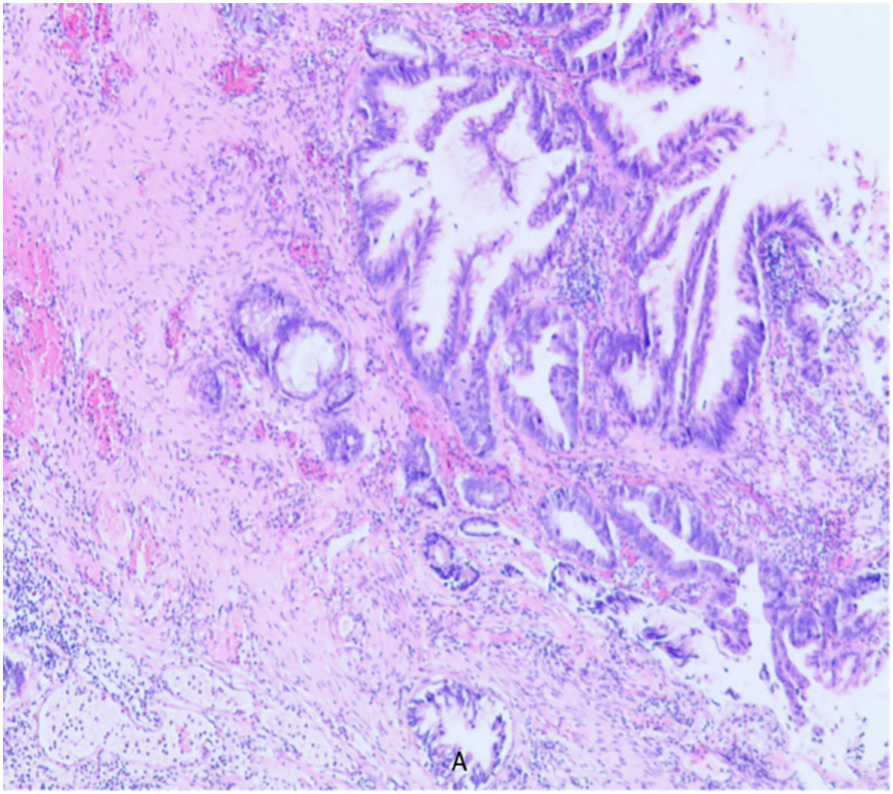
Adenocarcinoma (adenoidal arrangement of cancer cells).

A 79-year-old elderly male patient with no past medical history. He was first admitted to the hospital in 2019-December for progressive dysuria for two years, aggravated for four days and frequently accompanied by putrefactive material excreted with urine. Outpatient rectal examination: prostate III degree enlargement, loss of central sulcus, softer texture, no tenderness, no nodules. Further examination: blood PSA 0.167ng/ml; ultrasound: prostate size of about 49mm×45mm×40mm, full morphology, smooth peritoneum, internal echogenicity is not homogeneous, and there are many spots and stripes of liquid dark areas. After urination, the residual urine volume was about 175 ml, and the maximal force of the urethra muscle was about 90 cmH2O. The preoperative diagnosis was “urinary retention and prostatic hyperplasia”. Based on thorough evaluation, transurethral electrical prostatectomy and cystoscopy were performed under epidural anesthesia. The operation went smoothly, and there was no obvious mass in the bladder, but the prostate tissue was abnormal, with putrefaction and multiple flocculent necrosis. The patient recovered well after the operation, and the urinary catheter was removed six days after the operation, and urination was smooth. Postoperative pathology report (As shown in **Figure 1**): adenocarcinoma (adenoidal arrangement of cancer cells). No endocrine or other adjuvant treatment was performed after discharge from the hospital.

**Figure 2:**
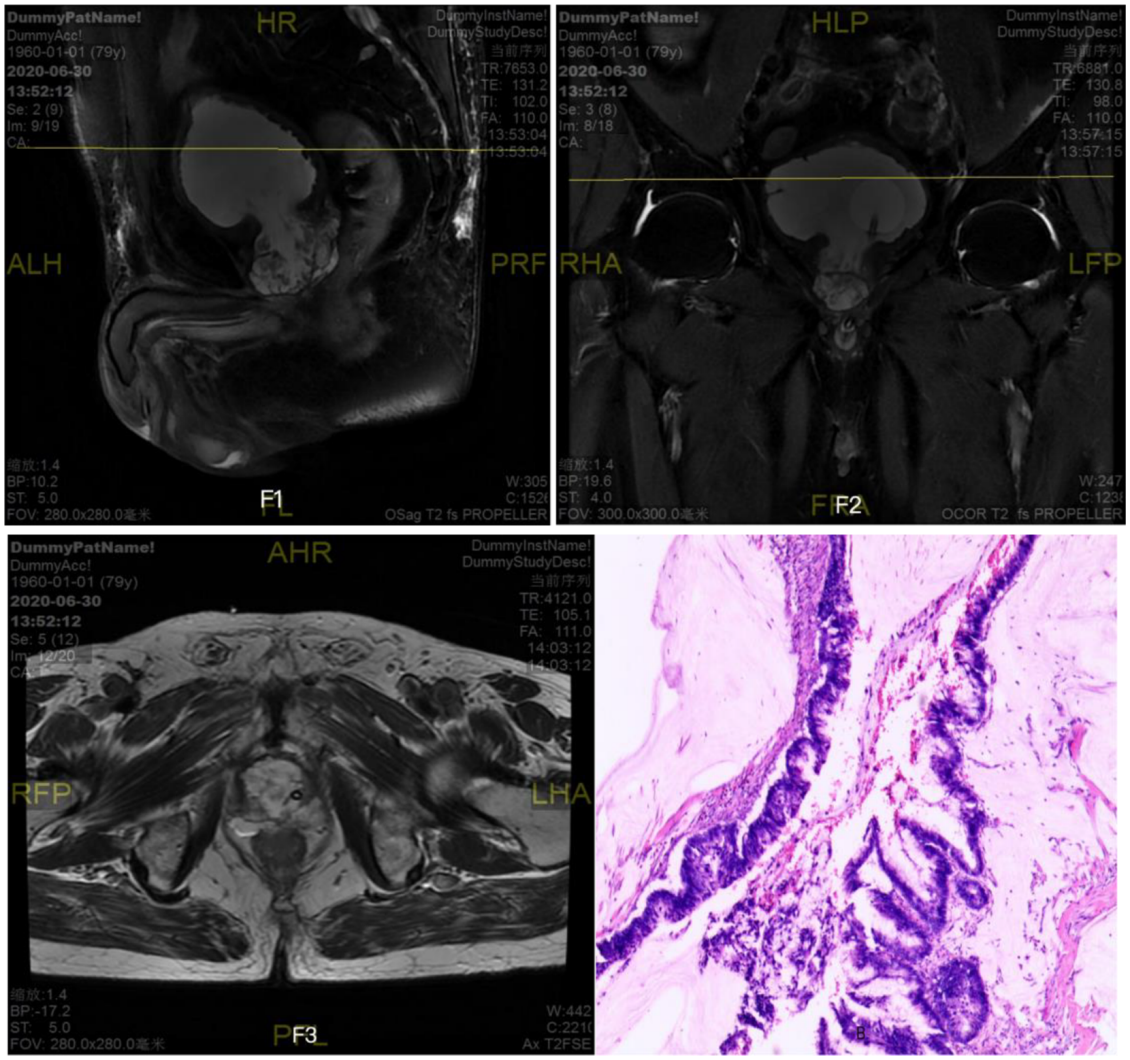
Prostate MRI (scanning)+prostate adenocarcinoma with mucinous carcinoma differentiation (cancer cells arranged in adenoid pattern and rich in mucus).

Six months later, the patient was admitted to the hospital again with no obvious cause for inability to urinate, accompanied by a painful sensation of lower abdominal distension, and the patient and his family members described that there was still a large amount of tofu dregs-like sediment flowing out of the urine during the six-month period after the operation. Subsequently, further relevant examinations were performed: blood PSA <0.003ng/ml; total testosterone: 340.19ng/dL; ultrasound: the size of the prostate was about 56mm×51mm×54mm; prostate MRI (scanning) (As shown in **Figures 2F1–F3**): the size of the prostate was enlarged, the morphology of the signal was distorted, and the bilateral peripheral bands were not clearly displayed, with multiple irregular signal shadows, low signal in T1WI, high signal in T2WI, slightly higher signal in DWI;PET/CT showed post-prostatectomy changes, no clear FDG metabolism increase foci were seen in the operation area, and no abnormality was seen in whole-body imaging; no abnormality was seen in whole-body skeletal imaging. Elective laparoscopic radical prostatectomy for prostate cancer was performed under general anesthesia, during which the prostate was completely removed and the operation went smoothly. Postoperative pathological return (As shown in **Figure 2**): prostate adenocarcinoma with mucinous carcinoma differentiation (cancer cells arranged in adenoid pattern and rich in mucus); immunohistochemistry showed: P504s (partially +), CK7 (+), CK20 (partially +), CDX2 (+), B-catenin (plasma, membrane +), PSA (9). Pathologic basal cut margins and tips were negative. The patient had an uneventful postoperative recovery and was discharged after half a month without subsequent adjuvant therapy such as medications. In addition, in order to exclude secondary mucinous adenocarcinoma, the patient underwent gastroscopy and colonoscopy 3 months after prostatectomy, and the results suggested chronic superficial gastritis.

**Figure 3:**
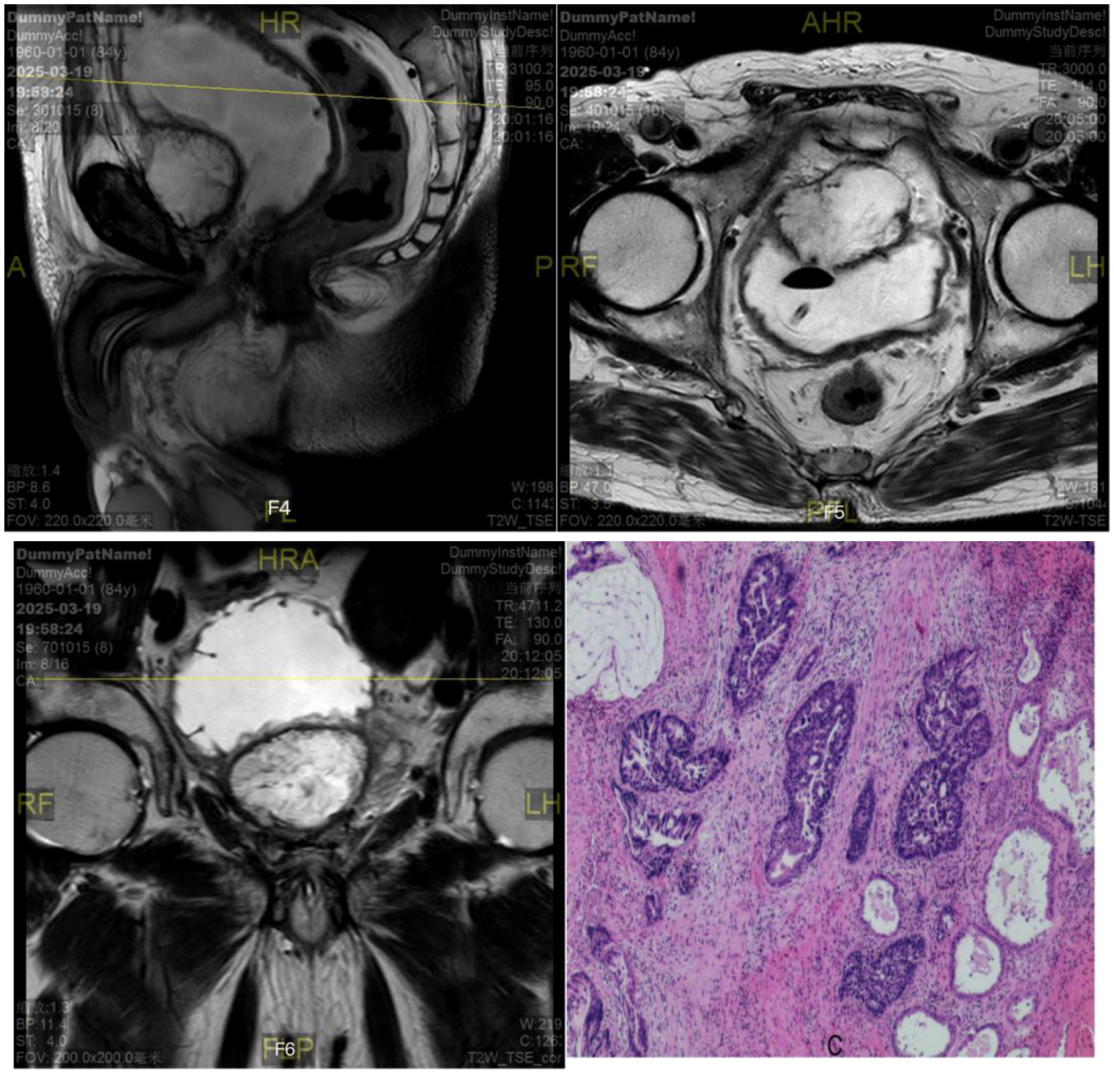
MRI of the prostate+the extraperitoneal mass was adenocarcinoma.

The patient’s white flocculent and sediment in urine gradually increased one year after the second operation, but no special intervention or treatment was performed, and he was re-admitted to the hospital in March 2025 because of the reappearance of symptoms of urinary difficulty for more than one month. Relevant examinations showed: blood PSA 0.520ng/ml; ultrasound of the urinary system: hypoechoic mass in the bladder; MRI of the prostate (As shown in **Figures 3F4–F6**): abnormal signal mass in the anterior and inferior part of the bladder; CT of the whole ureter: mass in the anterior part of the bladder after surgery for prostate cancer, and metastasis or recurrence of the tumor was considered; there was no abnormality in the whole body skeletal imaging. The preoperative diagnosis was “urinary retention, bladder tumor”. Due to the severe symptoms reported by the patient, laparoscopic resection of retroperitoneal lesion and laparoscopic partial cystectomy were performed under general anesthesia with tracheal intubation. During the operation, hard texture of the retropubic tissues was seen, with obvious adhesions, and after freeing, an irregular retropubic mass was seen with the size of about 4cm×4cm, which was not detachable from the bladder wall, and it was seen that the tumor encroached into the anterior wall of the bladder and formed a large quantity of mucus on the surface of mucous membrane of the anterior wall of the bladder. On re-exploration, a small amount of mass was seen in the internal urethral orifice, which invaded bilateral ureteral orifices. Considering the consequences of not being able to anastomose after resection, it was decided that the tumor would not be resected for the time being, and adjuvant radiotherapy would be given after the operation, which went smoothly, and the patient returned to the ward. In the ward, the patient had repeated bladder irrigation for several days due to repeated blockage of the indwelling urinary catheter by ureteric flocculent material. Postoperative pathological findings (As shown in **Figure 3**): the extraperitoneal mass was adenocarcinoma, partially associated with mucinous adenocarcinoma differentiation; a small amount of heterogeneous epithelium was seen in part of the bladder to consider adenocarcinoma involvement, and a small amount of acellular mucus was seen; immunohistochemistry results: CK7 (a small portion of +), CK20 (+), P504s (partially +), CKhigh (a small portion of +), KI67-MBI1 (about 80%), CDX2 (+), Villin (+), CK(+), B-catenin (membrane+), PSA (9). No concomitant treatment such as radiotherapy was performed after the operation and he is still being followed up.

## Discussion

The term mucinous adenocarcinoma should be used only when the amount of extracellular mucus is large enough to produce a pool of mucus, which shows that prostate mucinous adenocarcinoma accounts for only 0.2-0.4% of prostate cancers (4, 9–11). Epidemiology As mentioned above, prostate mucinous adenocarcinoma, as a specific type of prostate cancer, is characterized by a very low incidence, and its biological behavior is still unclear.The etiology of MC may be related to genetics, environment, androgens, etc. Lane et al. (9) counted 3613 patients with prostate cancer, and a total of 14 cases of prostate mucinous adenocarcinoma were found. Dhom et al. (3)suggested that the origin of prostate mucinous adenocarcinoma may be related to the endocrine epithelium of the prostate. The secretion of large amounts of mucus is a pathologic feature of prostate mucinous adenocarcinoma. Most of these tumor cells contain mucus-filled cystic or cyst-like structures, and the secretion of mucus in a non-expandable glandular lumen is not classified as mucinous adenocarcinoma. Histologically, they show extracellular lakes of mucus and common prostate adenocarcinoma structures. Papillary structures, cylindrical or goblet cells are rare, and indurated cells are occasionally found (12). Saito et al. (13)reported three types of prostatic mucinous carcinoma: (1) mucinous carcinoma; (2) mucinous carcinoma with imprinted cells: extracellular mucinous tissue exceeds 25% of the tumor cell volume, and imprinted cells are less than 25% of the tumor volume. (3) Indocyte carcinoma: the morphological structure of the indocytes accounted for at least 25% of the tumor cell volume.

Early prostate mucinous carcinoma is usually asymptomatic, but when the tumor invades or blocks the urethra or bladder neck, symptoms similar to lower urinary tract obstruction or irritation may occur, and in severe cases, acute urinary retention, hematuria, and incontinence may occur. Some statistics show that prostatic mucinous carcinoma can manifest as urinary obstruction (70.2%), hematuria (25.5%), and bladder irritation (17.0%) (14). MC is more likely to develop skeletal metastases, followed by lymph node, lung, liver, adrenal, renal, brain and diaphragmatic metastases, compared to follicular prostate cancer (15). And there was no difference in the metastatic pathways between follicular and mucinous prostate cancer (12). PSA may be elevated or normal in patients with mucinous prostate cancer, and elevated PSA levels are associated with bone metastasis in advanced stages of mucinous prostate cancer (1). It has been suggested that PSA may be elevated in about 77.8% of patients, and the degree of elevation is not significantly different from that of prostate follicular carcinoma (13). In immunohistochemical testing, immunostaining for PAP and PSA can be positive, with a 77.8% positive rate for PSA, while immunostaining for carcinoembryonic antigen (CEA) is negative. In our case, PSA was detected at low levels after three admissions, the first PSA test value was 0.167, and six months later PSA was lower than 0.003, so we considered that the normal prostate gland had been completely occupied by mucinous adenocarcinoma tissues and that there was no residual normal prostate tissue. Mucinous adenocarcinoma of the prostate is often predominantly sieve reticulum type in the mucus area, followed by gyriform tumor cell clusters, vesicles, or glandular structures (1), and mucinous adenocarcinoma of the prostate has a Gleason score of mostly 3 + 4, which is roughly equivalent to conventional adenocarcinoma of ISUP grade 4 in terms of biological behavior (3).There were no significant differences in age of onset, symptoms of dysuria, palpable hard nodules on anal fingerprinting, immunohistochemical staining for anti-PSA, or progression of the three different pathologic types of mucinous adenocarcinoma of the prostate. Differently, mucinous carcinomas had a higher percentage of elevated blood PSA, whereas the PSA was mostly normal in imprinted carcinomas and mucinous carcinomas containing imprinted cells (13).

The clinical manifestation of early prostate mucinous carcinoma is not typical, easily misdiagnosed as prostate hyperplasia, and the diagnosis can only be clarified after the return of pathological results, so the diagnosis of prostate mucinous carcinoma mainly relies on pathological diagnosis. Diagnostic criteria for diagnosing primary prostate cancer include: (1) the specimen section is wetter, with mucus sensation and grayish color; (2) the tumor cells secrete a large amount of acidic and neutral mucus stroma, and a large amount of extracellular mucus is seen microscopically to form a mucus paste and the total amount of mucus reaches or exceeds 25% of the tumor tissues; (3) the tumor cells originate in the epithelium of the prostate gland, prostatic tubule, or the capsule of the prostate gland;(4) Non-papillary tissues are long and resemble the pattern of glial carcinoma; (5) The possibility of mucinous carcinoma metastasis to tissues outside the prostate (mainly the digestive tract) has been ruled out (8, 14, 16). The cancerous tissue may be jelly-like to the naked eye, and a large amount of mucus paste is seen under the microscope; irregular nests of cancer cells are seen floating in some of the mucus paste, and the nests may be solid, sieve-like, or small vesicular. Mucinous adenocarcinoma of the prostate needs to be differentiated from primary adenocarcinoma of the urothelial prostate and metastatic carcinoma originating from the bladder and colorectum. Tumor cells in primary prostatic mucinous adenocarcinoma differ from those of the small intestine or bladder in being oval or rounded cuboidal, nonhypercolumnar, and absent of cup-shaped cells (8, 14, 17). In primary adenocarcinoma of the urethra in the prostate department, the typical glandular structure of follicular adenocarcinoma was not seen in the mucus, and immunohistochemical techniques also showed negative PSA and PSAP. Although it is more difficult to distinguish prostatic mucinous adenocarcinoma from invasive bladder adenocarcinoma in some cases (e.g., it may be positive for both PSAP), bladder adenocarcinomas are focally PSA- and PSAP-positive, whereas prostatic adenocarcinomas are diffusely positive. Mucinous adenocarcinoma of colon origin invades the prostate and microscopically appears as a lake of mucus mixed with or accompanied by vesicular tumor cells, clusters, or isolated cells. Tumor hyperplasia infiltrates prostate tissue. Colon-origin tumors are PSA-negative, CDX2- and CEA-positive or nuclear β-linker-positive and can also be differentiated by combining clinical data with a history of colon cancer or endoscopy (18).

No uniform standards and protocols have been established for the diagnosis and treatment of prostate mucinous adenocarcinoma. Treatment methods mainly include surgery, endocrine therapy, radiotherapy and combination therapy. The efficacy of these conventional treatment modalities does not differ from the outcome of treating common prostate cancer (19). For patients under 70 years of age, with a life expectancy greater than 10 years and in good health, radical prostatectomy for prostate cancer is of greater significance in improving the 5- and 10-year survival rates of patients (20). A study followed 47 patients with prostate mucinous carcinoma after radical surgery (34 patients with T1a stage, 7 patients with T2a stage, and 6 patients with T2b stage), and the average tumor progression-free survival was 5.6 years, and the median survival was 6 years (20). Patients with a survival time of 15 years after radical prostate mucinous adenocarcinoma exist in the world (6). For patients without an indication for radical surgical intervention, endocrine therapy may be chosen based on specific staging. Complete androgen blockade, i.e., depot therapy, is available, including surgical depot, pharmacologic depot (subcutaneous LHRHA), and oral androgen receptor blockers (5). The sensitivity of the three different pathologic types of mucinous carcinoma to endocrine therapy varies. Mucinous carcinomas were more sensitive to endocrine therapy (77.8%), whereas imprinted cell carcinomas and mucinous carcinomas containing imprinted cells were completely insensitive to endocrine therapy. Neoadjuvant hormone therapy may reduce recurrence in patients with mucinous carcinoma (12). Mucinous carcinoma of the prostate responds poorly to radiation therapy, and no studies of long-term follow-up after radiation or chemotherapy have been reported. In addition, for complications such as hematuria, urinary retention, hydronephrosis, and urinary stones, appropriate treatment can be given according to the condition. Mucinous adenocarcinoma of the prostate has faster oncologic progression and earlier distant metastasis than follicular adenocarcinoma of the prostate. Saito et al (13). concluded that the prognosis of mucinous adenocarcinoma of the prostate is similar to that of highly differentiated adenocarcinoma of the prostate, with a survival rate of about 50% at 3 years and about 25% at 5 years. In conclusion, radical prostatectomy should be performed for those with early confined and good general condition; for advanced prostatic mucinous adenocarcinoma of advanced age patients, surgery or drug depot, androgen receptor blocker and other treatments are feasible, which can be supplemented with high-energy ultrasound focusing and radiotherapy. Early diagnosis and accurate clinical staging of prostate mucinous adenocarcinoma are extremely important for the formulation of reasonable treatment plan. In this case, the patient had a low PSA level and was considered insensitive to endocrine therapy, so endocrine treatment was not recommended for the time being. Given the high Ki-67 index, the tumor was deemed aggressive, and postoperative adjuvant radiotherapy was advised. However, during follow-up, the patient and their family declined radiotherapy due to the patient’s age and body mass considerations.

## Data Availability

The original contributions presented in the study are included in the article/Supplementary Material. Further inquiries can be directed to the corresponding author.
